# Current antibiotic and prophylactic antifungal drug policies in UK neonatal intensive care units: a national survey

**DOI:** 10.1093/jacamr/dlaf194

**Published:** 2025-10-16

**Authors:** Carla Kantyka, Rishini Wanigasekara, Vennila Ponnusamy, Paul T Heath, Paul Clarke

**Affiliations:** Neonatal Intensive Care Unit, Norfolk and Norwich University Hospitals NHS Foundation Trust, Colney Lane, Norwich NR4 7UY, UK; Neonatal Intensive Care Unit, Ashford and St Peter’s NHS Foundation Trust, Guildford Road, Chertsey KT16 0PZ, UK; Neonatal Intensive Care Unit, Ashford and St Peter’s NHS Foundation Trust, Guildford Road, Chertsey KT16 0PZ, UK; Vaccine Institute, Centre for Neonatal and Paediatric Infection, City St George’s, University of London, Cranmer Terrace, London SW17 0RE, UK; Neonatal Intensive Care Unit, Norfolk and Norwich University Hospitals NHS Foundation Trust, Colney Lane, Norwich NR4 7UY, UK; Norwich Medical School, University of East Anglia, Norwich NR4 7TJ, UK

## Abstract

**Objectives:**

To survey the current antibiotic and antifungal drug practices of UK neonatal intensive care units (NICUs), and to identify antibiotic preferences and policies for treatment of early- and late-onset sepsis (EoS and LoS), meningitis, and antifungal prophylaxis.

**Methods:**

Between January and May 2024, we contacted all 53 tertiary-level UK NICUs via telephone and/or e-mail. We requested a copy of each unit’s guidelines for antibiotic treatment of EoS and LoS, and antifungal prophylaxis.

**Results:**

We obtained guidelines from 53/53 (100%) units. A penicillin and aminoglycoside combination was the consistent first-line recommendation for EoS in 51/53 (96%) units. Only a minority (11/53; 21%) units specified any second-line antibiotic regimen for EoS, though most (44/53; 83%) specifically recommended amoxicillin for suspected listeriosis. For LoS, almost all NICUs (52/53; 98%) provided specific guidance on empirical first-line antibiotic treatment, with empirical narrow-spectrum antibiotics as first-line LoS treatment for term neonates in 42/53 (79%) NICUs and for preterm neonates in 41/53 (77%) NICUs. Fifty-four percent (29/53) of units included specific LoS recommendations for neonates with indwelling central venous catheters. Sixty-six percent (35/53) of NICUs included cefotaxime in their empirical meningitis regimens. Eighty-five percent (45/53) of units had clear guidelines for antifungal prophylaxis.

**Conclusions:**

While EoS treatment was consistent across units, there remained wide variation in antibiotic regimens used for LoS and meningitis, and for neonates with indwelling central venous catheters. Guidelines specific to preterm neonates were limited. The practice of routine antifungal prophylaxis has been more prevalent since the last UK survey in 2006–07 but is still neither universal nor consistent.

## Introduction

In March 2024, the UK’s National Institute for Health and Care Excellence (NICE) issued updated guidelines recommending better antibiotic targeting for those at highest risk of suspected sepsis, to ensure timely treatment to the right patients, while avoiding overuse of antibiotics, which can lead to antibiotic resistance.^1^ The neonatal population comprises a most vulnerable patient age group with one of the highest rates of antibiotic usage. With recent publication of the British Association of Perinatal Medicine (BAPM) framework for management of extreme preterm neonates,^2^ increasing numbers of lower gestation and extremely low-birth-weight neonates are now being cared for, with a correspondingly high associated rate of antibiotic use due to their high risk of morbidity and mortality from sepsis.^3,4^

Recent years have seen an increase in multi-drug resistant pathogens in preterm neonates,^5,6^ emphasizing that rigorous antibiotic stewardship is more crucial than ever before. The last UK survey of antibiotic practices in neonatal units was conducted in 2006–07.^7^ Since then, NICE has published and revised its neonatal infection guidelines.^8^ What is unclear is how UK antibiotic stewardship in neonatal ICUs (NICUs) has evolved since then.

Therefore, to better understand current practice and trends, we reviewed guidelines from all UK tertiary-level NICUs and compared these practices against current NICE recommendations for treatment of early- and late-onset sepsis (EoS and LoS), and for antifungal prophylaxis.^8^

## Methods

Between January 2024 and May 2024, we contacted all UK NICUs via telephone and/or e-mail. NICUs, also referred to as Level 3 Units, look after neonates needing the highest level of medical and (in some units) surgical care, and neonates who do not fit the criteria for Local Neonatal Unit or Special Care Unit admission. Neonates admitted to NICUs are usually born before 28 weeks’ gestation and/or are very sick or require surgery. The list of the UK’s 53 NICUs was derived from the websites of the 13 UK Neonatal Networks on the BAPM home page.^9^ We requested either the governance lead or a senior medical staff member at each unit to consent to share with us a copy of their unit’s antibiotic treatment and antifungal prophylaxis guidelines. We reviewed these guidelines to assess antibiotic recommendations for EoS, LoS, antifungal prophylaxis policies, and some specific clinical situations. Our findings were evaluated in comparison with those of the previous national UK survey of neonatal unit antibiotic policies conducted in 2006–07, assessed using similar proforma headings,^7^ and also evaluated against the recently updated 2021 NICE guideline ‘Neonatal infection: antibiotics for prevention and treatment’.^8^ The latter specifies the use of intravenous (IV) benzylpenicillin with gentamicin as the first-choice antibiotic regimen for empirical treatment of suspected EoS, unless microbiological surveillance data show local bacterial resistance patterns that indicate the need for a different antibiotic. For neonates with suspected late-onset neonatal infection who are already in a neonatal unit, they specify a combination of narrow-spectrum antibiotics as first-line treatment and again to use local antibiotic susceptibility and resistance data (or national data if local data are inadequate) when deciding which antibiotics to use.

For the purpose of this study, we defined narrow-spectrum antibiotics as being those that act against a limited range of bacteria, usually targeting either mainly Gram-positive or mainly Gram-negative organisms. For example, we classed benzylpenicillin and the aminoglycosides gentamicin and amikacin as narrow-spectrum antibiotics.

We also assessed antifungal prophylaxis policies against the NICE guidelines, which recommend routine antifungal prophylaxis for very low-birth-weight neonates (birth weight <1500 g) or less than 30 weeks’ gestational age when receiving antibiotic treatment for LoS. According to the NICE guidelines, oral nystatin is the preferred option over IV fluconazole unless the oral route of administration is not possible.

As an anonymized evaluation surveying current clinical practices and policies, formal research ethics approval was not necessary for this study, and this was confirmed in writing by the research services manager of the Norfolk and Norwich University Hospital.

## Results

We obtained responses from 53/53 (100%) NICUs; all provided a copy of their unit guidelines for our evaluation.

### EoS: first-line antibiotics

Table 1 summarizes antibiotic choices for EoS. Benzylpenicillin and an aminoglycoside combination was the primary antibiotic regimen chosen in 51/53 (96%) of the units for treating suspected EoS. The remaining 2/53 (4%) units used cefotaxime monotherapy for EoS. Gentamicin was the most frequently used aminoglycoside, in 45/53 (85%) units across all gestations, in line with the NICE recommendation.^8^ Of note, of the two units that used cefotaxime monotherapy as first line for EoS, one had a second-line EoS policy, which recommended benzylpenicillin and gentamicin, while the other did not specify any second-line regimen. Additionally, 44/53 (83%) units recommended including amoxicillin when listeriosis was suspected clinically, in line with NICE guidelines, while 9/53 (17%) unit guidelines did not contain any specific recommendation for covering listeriosis.

**Table 1.** First- and second-line antibiotic policies for early onset sepsis among 53 UK NICUs

	Antibiotic regimen	*n* (%) units
EoS: first line	benzylpenicillin and gentamicin	45 (85)
	benzylpenicillin and amikacin	5 (9)
	benzylpenicillin and gentamicin or cefotaxime	1 (2)
	cefotaxime (monotherapy)	2 (4)
EoS: second line	none specified	42 (79)
	cefotaxime (monotherapy)	2 (4)
	cefotaxime and gentamicin	2 (4)
	cefotaxime and amoxicillin	1 (2)
	cefotaxime and benzylpenicillin and gentamicin	1 (2)
	benzylpenicillin and gentamicin	1 (2)
	piperacillin-tazobactam and vancomycin	1 (2)
	piperacillin-tazobactam/teicoplanin/flucloxacillin and amikacin	1 (2)
	meropenem and vancomycin	1 (2)
	meropenem (monotherapy)	1 (2)

### EoS: second-line antibiotics

Only 11/53 (21%) units specified a second-line antibiotic regimen for EoS, and those that did showed wide variation in choice of agents (Table 1). Of these 11, only 6 (11% of overall NICUs) recommended cefotaxime, 3 (6% of overall NICUs) recommended penicillin, and 2 (4% of overall NICUs) recommended meropenem. The remaining 42/53 (79%) units did not have any second-line antibiotic recommendation. Of the six units recommending cefotaxime as their second-line EoS regimen, two used it as monotherapy and four used it in conjunction with one or more other agents. Overall, there was no notable difference in EoS antibiotic choices between term and preterm neonates.

### LoS: first-line antibiotics

Almost all NICUs (52/53; 98%), provided specific guidance for empirical first-line LoS antibiotic treatment (Table 2). There were 15 unique regimens adopted by these 52 neonatal units. Narrow-spectrum antibiotics, as recommended by NICE as first-line LoS treatment,^8^ were included in regimens for term neonates in 42/53 (79%) NICUs and for preterm neonates in 41/53 (77%) NICUs.

**Table 2.** LoS treatment in term and preterm neonates: first- and second-line antibiotic policies in 53 UK NICUs according to presence or absence of CVCs

LoS without CVC: first-line antibiotic regimen for both term and preterm neonates^a^		LoS without CVC: second-line antibiotic regimen for both term and preterm neonates	
Flucloxacillin and gentamicin	28 (53)	No regimen specified	17 (32)
Vancomycin and gentamicin	5 (9)	Meropenem	9 (17)
Piperacillin/tazobactam and vancomycin^a^	3 (6)	Vancomycin and gentamicin	5 (9)
Flucloxacillin and amikacin	4 (8)	Cefotaxime and vancomycin	2 (4)
Teicoplanin and gentamicin	2 (4)	Meropenem and vancomycin	2 (4)
Amikacin and piperacillin/tazobactam	2 (4)	Piperacillin/tazobactam and amikacin	1 (2)
Benzylpenicillin and gentamicin^a^	1 (2)	Flucloxacillin, gentamicin and cefotaxime	1 (2)
Co-amoxiclav and gentamicin	1 (2)	Flucloxacillin and gentamicin	1 (2)
Ceftazidime and vancomycin	1 (2)	Vancomycin and piperacillin/tazobactam	1 (2)
Flucloxacillin and a choice of gentamicin, amikacin or vancomycin	1 (2)	Amikacin and meropenem	1 (2)
Cefotaxime and teicoplanin	1 (2)	Vancomycin and ceftazidime	1 (2)
Flucloxacillin, gentamicin and amoxicillin	1 (2)	Teicoplanin and meropenem	1 (2)
Cefotaxime	1 (2)	Piperacillin/tazobactam	1 (2)
Cefotaxime and gentamicin	1 (2)	Flucloxacillin, gentamicin and ciprofloxacin	1 (2)
None specified	1 (2)	Vancomycin, cefotaxime and metronidazole	1 (2)
		Flucloxacillin and cefotaxime	1 (2)
		Meropenem and gentamicin	1 (2)
		Piperacillin/tazobactam and vancomycin	1 (2)
		Teicoplanin and gentamicin	1 (2)
		Ceftazidime and vancomycin	1 (2)
		Cefotaxime and gentamicin	1 (2)
		Vancomycin and amikacin	1 (2)
		Vancomycin and meropenem	1 (2)

First-line regimen with indwelling CVC for both		Second-line regimen with indwelling CVC for both	
term and preterm neonates		term and preterm neonates	
Vancomycin and gentamicin	13 (24)	No agent specified	16 (30)
Flucloxacillin and gentamicin	9 (17)	Vancomycin and meropenem	10 (18)
Cefotaxime and vancomycin	7 (13)	Meropenem	8 (15)
Teicoplanin and gentamicin	4 (8)	Vancomycin and gentamicin	5 (9)
Piperacillin/tazobactam and vancomycin	6 (11)	Teicoplanin and gentamicin	2 (4)
Flucloxacillin and amikacin	2 (4)	Piperacillin/tazobactam and vancomycin	3 (6)
Vancomycin and ceftazidime	2 (4)	Adding in gentamicin	1 (2)
Flucloxacillin and gentamicin OR amikacin and vancomycin	1 (2)	Flucloxacillin and gentamicin	1 (2)
Teicoplanin and cefotaxime	1 (2)	Cefotaxime and vancomycin	1 (2)
Piperacillin/tazobactam, vancomycin and gentamicin	1 (2)	Flucloxacillin and gentamicin and ciprofloxacin	1 (2)
Teicoplanin and amikacin	1 (2)	Piperacillin/tazobactam OR teicoplanin AND amikacin	1 (2)
Vancomycin AND gentamicin OR cefotaxime	1 (2)	Ceftazidime and vancomycin	1 (2)
Ceftazidime and teicoplanin	1 (2)	Vancomycin and amikacin	1 (2)
Benzylpenicillin OR flucloxacillin AND gentamicin	1 (2)	Teicoplanin and meropenem	1 (2)
Co-amoxiclav and gentamicin	1 (2)	Vancomycin and cefotaxime and metronidazole	1 (2)
Ceftazidime AND vancomycin OR vancomycin AND amikacin	1 (2)		
None specified	1 (2)		

Data are *n* (%); CVC, central venous catheter.

^a^Guidance for preterm versus term babies differed in one instance only—in a single centre where piperacillin/tazobactam and vancomycin was recommended for preterm infants in lieu of benzylpenicillin and gentamicin.

The combination of flucloxacillin and an aminoglycoside was the most common first-line choice: in 32/53 (60%) NICUs for term, and 30/53 (56%) NICUs for preterm neonates. Other agents paired with an aminoglycoside included glycopeptides (e.g. vancomycin or teicoplanin) in 12/53 (23%) NICUs, and broad-spectrum penicillins (e.g. piperacillin/tazobactam or co-amoxiclav) in 4/53 (7%) NICUs.

The number of units recommending an aminoglycoside as part of their first-line LoS treatment regimen was 43/53 (81%) for preterm neonates and 46/53 (86%) for term neonates. The only two aminoglycosides included among all the guidelines were gentamicin and amikacin. Gentamicin was included far more frequently than amikacin, with 39/46 (84%) regimens specifying gentamicin for term and 37/43 (86%) for preterm neonates, and the remaining units specifying amikacin.

Specific anti-coagulase-negative staphylococcus (CoNS) antibiotic cover (such as vancomycin or teicoplanin) was not included as part of their first-line regimen for LoS for term neonates in 40/53 (75%) NICUs or for preterm neonates in 39/53 (73%) NICUs. Only 1/53 (2%) NICUs had a different first-line LoS regimen for term versus preterm neonates.

### LoS: second-line antibiotics

Empirical second-line antibiotics for LoS were specified in 36/53 (68%) NICUs, but the agents varied significantly, with the most common second-line regimen being meropenem monotherapy in 9/53 (17%) followed by vancomycin and gentamicin in 5/53 (9%) NICUs (Table 2). Other recommendations included a mixture of single agents and combination agents, for example teicoplanin, piperacillin/tazobactam, ceftazidime, co-amoxiclav and amikacin (Table 2).

### Neonates with indwelling central venous catheters (CVCs)

Overall, 52/53 (98%) of units made specific mention in their guidelines of antibiotic treatment for neonates with indwelling CVCs. Of those, 39/52 (75%) units included CoNS cover with either vancomycin or teicoplanin, implying that in 14/53 (26%) units the use of CoNS-specific antibiotic cover would be delayed. The most widely used single-agent regimen was vancomycin as monotherapy in 32/53 (60%) units for neonates with a CVC *in situ*, while vancomycin was used in combination with gentamicin in 10/53 (18%) units. The choice of antibiotics specified for neonates with indwelling CVCs did not differ between term and preterm neonates in any unit.

### Empirical antibiotic therapy for meningitis

The most common regimen of choice for covering suspected meningitis was amoxicillin and cefotaxime, used in 20/53 (38%) units. The next most common regimens were gentamicin and cefotaxime, used in 8/53 (15%) units, and cefotaxime as monotherapy in 7/53 (13%) units. No unit specified meropenem monotherapy for treating meningitis. Overall, 35/53 (62%) units included cefotaxime in their empirical meningitis regimen.

Our assessment of treatment for confirmed meningitis was based on review of the 49/53 (92%) antibiotic guidelines that specifically incorporated meningitis management. Sixty-seven percent (33/49) of these units recommended a minimum 14 day treatment for confirmed group B *Streptococcus* meningitis. Thirty-five percent (17/49) of units recommended a minimum 21 day course for *Listeria* meningitis. Only 1/49 (2%) had any specific recommendations for meningitis in extremely preterm neonates—which was to add a single dose of amikacin. Ninety-eight percent (48/49) of unit guidelines did not differentiate between preterm and term neonates for management of confirmed meningitis in respect of either antibiotic choices or treatment duration.

### Antifungal prophylaxis

Clear written guidelines for the use of routine antifungal prophylaxis was provided by 45/53 (85%) of the units, whilst 8/53 (15%) had no specific guidelines for antifungal prophylaxis. Definition of a ‘high-risk neonate’ for routine antifungal prophylaxis varied widely between NICUs in terms of birth-weight and gestational-age cut-offs. Just under one-third of units, (16/53; 30%) followed the specific NICE recommendations regarding stated cut-offs.^8^ Among the NICUs surveyed, we found birth gestational-age thresholds for antifungal prophylaxis varied from <25 weeks to <32 weeks and birth-weight thresholds varied from <750 g to <1500 g. Overall, 32/53 (60%) units considered other relevant criteria to indicate antifungal prophylaxis, including concomitant central catheters, ventilatory support and abdominal pathology. Specifically, 4/53 (8%) units considered suspected necrotizing enterocolitis as an indication to initiate antifungal prophylaxis.

Oral nystatin was recommended in 11/53 (21%) units; 19/53 (36%) recommended oral nystatin or IV fluconazole depending on oral tolerance, gestational age and birth weight. Despite the NICE recommendations of oral nystatin as first line, almost one-third of units (15/53; 28%), recommended IV fluconazole for prophylaxis instead, with some advising to switch to oral fluconazole when able.

## Discussion

We have evaluated the antibiotic recommendations of all 53 UK NICUs and compared them against the recommendations from the recently updated 2021 NICE guidelines,^8^ and with the findings of the last national antibiotic survey of neonatal units in the UK and Republic of Ireland done in 2006–07.^7^ A major strength of our study is that we had responses from 100% of NICUs, allowing a meaningful interpretation of current practices for the highest-risk neonates.

Assessed against the 2021 NICE guideline recommendations, we found that significantly more units now have compliant EoS antibiotic policies, 96% in the present survey compared with 69% overall in 2006–07 (unadjusted by care level of neonatal unit).^7^ Additionally, more NICUs now include a penicillin in first-line EoS policies, 96% in 2024 compared with 90% in 2006–07,^7^ an improved rate of coverage for *Listeria monocytogenes*, and in line with the March 2024 update of the NICE guidelines.^8^ We note that there is no recommendation in the NICE guideline for second-line antibiotic choices for EoS and, indeed, the agent of choice varied widely between units.

Most NICUs (96%; 51/53) are now using narrow-spectrum first-line agents for LoS in line with the NICE guideline, an improved rate compared with that (69%) reported in the 2006–07 survey.^7^ However, for second-line regimens and for neonates with and without CVCs we found little consensus. It is perhaps surprising that 14/53 (26%) of NICUs did not have specific recommendations to include a glycopeptide antibiotic among the first-line antibiotic treatment regimen for LoS for very preterm neonates with indwelling CVCs, particularly as CoNS account for the majority of catheter-associated sepsis episodes in such neonates,^4^ and this may inevitably leave some vulnerable. Yet it is also notable that the current NICE guideline does not include specific antibiotic recommendations for such neonates either. Reflecting this, we found little difference in guidelines for treating extremely preterm neonates compared with term neonates across all units surveyed.

The March 2024 update to the NICE ‘Neonatal infection’ guideline included recommendations for treating early- and late-onset neonatal meningitis in neonatal units.^8^ The empirical combination of cefotaxime and amoxicillin is recommended, so it is of concern that only just over half of NICUs (29/53; 55%) had specific guidelines to provide this combination therapy for suspected meningitis. Although cefotaxime was included in an empirical regimen in 35/53 (66%) NICUs, the implication remains that for a significant proportion of neonates with bacterial meningitis, optimal therapy will be delayed – a risk factor for poor outcomes.^10^

In comparison with the 2006–07 survey, which showed that only 32% of neonatal units overall had a fungal prophylaxis policy,^7^ our present survey has shown that 85% of NICUs now have formal guidelines for routine antifungal prophylaxis. While this is a positive step, despite the clear 2021 NICE guidance there remains little consensus between units in terms of antifungal agents, route of administration, and which risk factors are critical. At-risk neonates cared for in units without formal guidelines and who do not receive routine antifungal prophylaxis may be at an increased risk of invasive fungal infections and significant associated morbidity due to non-compliance with current NICE recommendations.

A limitation of our study is that its scope did not include qualitative inquiry into reasons behind local choices and preferences where there was deviation from the current national recommendations. This issue is worthy of further study.

In summary, our current and comprehensive UK survey of antibiotic and antifungal policies of tertiary-level NICUs has shown more consistent policies regarding management of EoS, LoS and antifungal prophylaxis than was found in previous surveys. While practices appear more aligned with the updated 2021 NICE guidance, we believe there is still scope for further updates to the guideline to ensure optimal and standardized antibiotic and antifungal cover for the highest-risk neonates, to improve clinical outcomes, and to prevent antibiotic resistance. We encourage units to use the results of this survey as the basis for a review of their own guidelines and practice, including a discussion of where they differ from national guidance. We also encourage them to undertake prospective clinical audits of antibiotic use, because clinical practices do not always comply with written policies.^11^ Finally, we highlight the need for future qualitative research to understand how antibiotic choices are made, especially where they deviate from national guidelines.
